# Spinach and Chive for Kidney Tubule Engineering: the Limitations of Decellularized Plant Scaffolds and Vasculature

**DOI:** 10.1208/s12248-020-00550-0

**Published:** 2020-12-28

**Authors:** Katja Jansen, Marianna Evangelopoulou, Carla Pou Casellas, Sarina Abrishamcar, Jitske Jansen, Tina Vermonden, Rosalinde Masereeuw

**Affiliations:** 1 Division of Pharmacology, Utrecht Institute for Pharmaceutical Sciences, Utrecht University, Utrecht, The Netherlands; 2 Department of Pathology, Radboud Institute for Molecular Life Sciences, Radboud university medical center, Nijmegen, The Netherlands; 3 Department of Pediatric Nephrology, Radboud Institute for Molecular Life Sciences, Radboud university medical center, Amalia Children’s Hospital, Nijmegen, The Netherlands; 4 Division of Pharmaceutics, Utrecht Institute for Pharmaceutical Sciences, Utrecht University, Utrecht, The Netherlands; 5 Division of Pharmacology, Utrecht Institute for Pharmaceutical Sciences, Universiteitsweg 99, 3584 CG Utrecht, The Netherlands

**Keywords:** Tissue engineering, Regenerative medicine, Proximal tubule, Plant scaffolds, Decellularization

## Abstract

Tissue decellularization yields complex scaffolds with retained composition and structure, and plants offer an inexhaustible natural source of numerous shapes. Plant tissue could be a solution for regenerative organ replacement strategies and advanced *in vitro* modeling, as biofunctionalization of decellularized tissue allows adhesion of various kinds of human cells that can grow into functional tissue. Here, we investigated the potential of spinach leaf vasculature and chive stems for kidney tubule engineering to apply in tubular transport studies. We successfully decellularized both plant tissues and confirmed general scaffold suitability for topical recellularization with renal cells. However, due to anatomical restrictions, we believe that spinach and chive vasculature themselves cannot be recellularized by current methods. Moreover, gradual tissue disintegration and deficient diffusion capacity make decellularized plant scaffolds unsuitable for kidney tubule engineering, which relies on transepithelial solute exchange between two compartments. We conclude that plant-derived structures and biomaterials need to be carefully considered and possibly integrated with other tissue engineering technologies for enhanced capabilities.

## Introduction

To mimic native tissue, organ-specific cells are usually seeded onto or into scaffolds with a defined 3D structure and stimulated with growth factors, physicochemical factors, and perfusion. To date, cornea, skin, and articular joint tissue are among the most advanced engineered tissue constructs due to their relatively simple architecture (1). In contrast, the kidney is one of the most complex ones; structural and functional complexity make it impossible to recapitulate its original architecture with traditional top-down approaches (*e.g.*, micro-molding or 3D bioprinting) (2). The explosive advancement of organoid technology, a bottom-up approach in regenerative medicine, reflects our dependency on the power of nature: the most sophisticated tissues currently created in a dish are the result of cellular self-organization (3, 4). However, despite being complex in structure, kidney organoids are still immature and limited in size, and they lack a functioning drainage system. An intermediate tissue engineering approach is the de- and recellularization of existing tissue, which yields whole organ scaffolds with intact extracellular matrix (5–7). In a proof-of-concept study, recellularized rat kidneys were able to regenerate partial excretory functionality upon recellularization and experimental orthotopic transplantation (8, 9). For clinical consideration, however, such system has to be upscaled and optimized further, and also for advanced *in vitro* modeling, whole scaffolds are no sustainable option, except when cut into multiple scaffold slices (7). In 2017, Gershlak* et al*. drew attention to the concept of *crossing kingdoms* in tissue engineering by using plant-derived decellularized scaffolds for the fabrication of lab-grown organs with perfusable vasculature (10). In this and another follow-up study, the research group showed successful decellularization of various plant leaves and roots (*i.e*.*,* spinach, parsley, *Artemisia annua* leaves, *anthurium waroqueanum*, *Calathea zebrina*, bamboo, orchid, vanilla, and peanut hairy roots); recellularization was proven with several human cell sources (*i.e.,* human umbilical vein endothelial cells, dermal fibroblasts, mesenchymal stem cells, and pluripotent stem cell-derived cardiomyocytes) (10, 11). In fact, the concept of using decellularized cellulose for cell cultures has been developed earlier with apple hypanthium, for which cell adhesion, invasion, and proliferation have been shown with NIH3T3 fibroblasts, mouse C2C12 muscle myoblasts, and human HeLa epithelial cells. Moreover, biocompatibility and active blood vessel formation have been demonstrated after implantation *in vivo* (12, 13). Here, we adopted and evaluated the *crossing kingdoms* principle for *in vitro* modeling of kidney tubules. These studies showed that various mammalian cell types attach and grow on plant-derived cellulose scaffolds, but we want to stress that tissue-specific requirements must be considered. For instance, to facilitate active secretion of metabolic end products and drugs, engineered kidney tubules require a thin and small-sized tubular scaffold that separates the inner and outer compartment through a monolayer of kidney proximal epithelial tubular cells, preferably with drainage capacity. For this, we hypothesized that the vasculature of decellularized spinach leaves or hollow chive stems could serve as such scaffolds and examined them for functional requirements.

## Methods

### Scaffold Decellularization

Spinach leaves and chive stems, obtained from a local store, were placed in n-hexane on a shake rocker for 30 min to remove the cuticle. After rinsing with distilled water, they were placed on a perfusion pump or the shake rocker with daily refreshed 5% (v/v) sodium dodecyl sulfate (SDS) for 7 days. In parallel, a freshly isolated kidney from a healthy male surplus Wistar rat was perfused using a 5-day 1% (v/v) SDS and 30-min 1% (v/v) Triton X-100 decellularization protocol as described earlier (14). Following the protocol for plant tissue after 5% (v/v) SDS treatment, all plant samples were first washed with 10% (v/v) sodium chlorite and 1% (v/v) Triton-X100 for 5 days and ultrapure water for up to 7 days until samples were translucent. Until recellularization, plant samples were stored in 70% ethanol at room temperature, whereas the decellularized rat kidney was frozen at -20 °C.

### Scaffold Recellularization

Before recellularization, the samples were sterilized with 365 nm UV light (2.6 mW cm − 2, UVP CL-1000 for 15 min) and washed with sterile Hanks’ Balanced Salt Solution (HBSS) overnight. Kidney tissue was sliced into 150 μm pieces and sterilized with 0.2% (v/v) peracetic acid in 4% (v/v) ethanol for 10 min (7). While adhesive coating was unnecessary for kidney tissue, decellularized plant samples were coated for 4 h with 2 mg/mL l-3,4-dihydroxyphenylalanine (L-DOPA, Sigma Aldrich) dissolved in 10 mM Tris buffer at pH 8.5, which was pre-incubated at 37 °C for 45 min and 0.2 μm filter-sterilized (15). After thorough sample washing with HBSS, 500.000 parent or tetramethylrhodamine-isothiocyanate (TRITC)-labeled conditionally immortalized proximal tubule epithelial cells (ciPTEC) were seeded onto a rat kidney slice in an ultra-low attachment plate or injected into the spinach petiole or chive stem using a 22 gauge injection needle and ciPTEC medium composed as described earlier (7, 16, 17). The ciPTEC cell line was obtained as described by Wilmer *et al*. with informed consent of the donor and in accordance with the approved guidelines of the Radboud Institutional Review Board. In short, cells exfoliated in the urine of a healthy volunteer were transfected with SV40T and hTERT vectors and subcloned for a homogeneous cell population (16). Chive was turned 180° after 1 h and after 2 h; all recellularized samples were submerged in medium and cultured until confluency (16).

### Protein Quantification

Protein quantification was performed with the BCA Protein Assay Kit (Pierce Biotechnology) according to the manufacturer’s instructions, and plates were read at 595 nm using the iMark Microplate Absorbance Reader (Bio-Rad).

### Microscopic Imaging

Before DAPI staining (1:1000, Sigma-Aldrich), cells were fixed with 2% *w*/*v* paraformaldehyde in HBSS and permeabilized with 0.3% *v*/v triton X-100 in HBSS for 10 min (Sigma-Aldrich). Microscopic images were taken with a Keyence BZ-9000 fluorescence microscope or confocal microscope Leica TCS SP8 X.

### Mechanical Analysis

The mechanical behavior of decellularized chive was tested under uniaxial tensile loading using a Dynamic Mechanical Analyzer (DMA Q800, TA Instruments). Strain and stress at break were determined from the stress–strain curves.

### Diffusion Assay

A leakage assay was performed in custom-printed flow chambers (Ultimaker 3) on dialysis fibers (MicroPES type TF10 hollow fibers, Membrana GmbH, Germany) and decellularized chive, with 0.1 mg/ml fluorescein isothiocyanate-inulin (inulin-FITC, Sigma Aldrich) perfusion for 10 min. All results were statistically analyzed using one-way ANOVA (GraphPad Prism 8).

## Results

### Successful Application of De- and Recellularization Techniques on Spinach Leaves and Chive

**Fig. 1:**
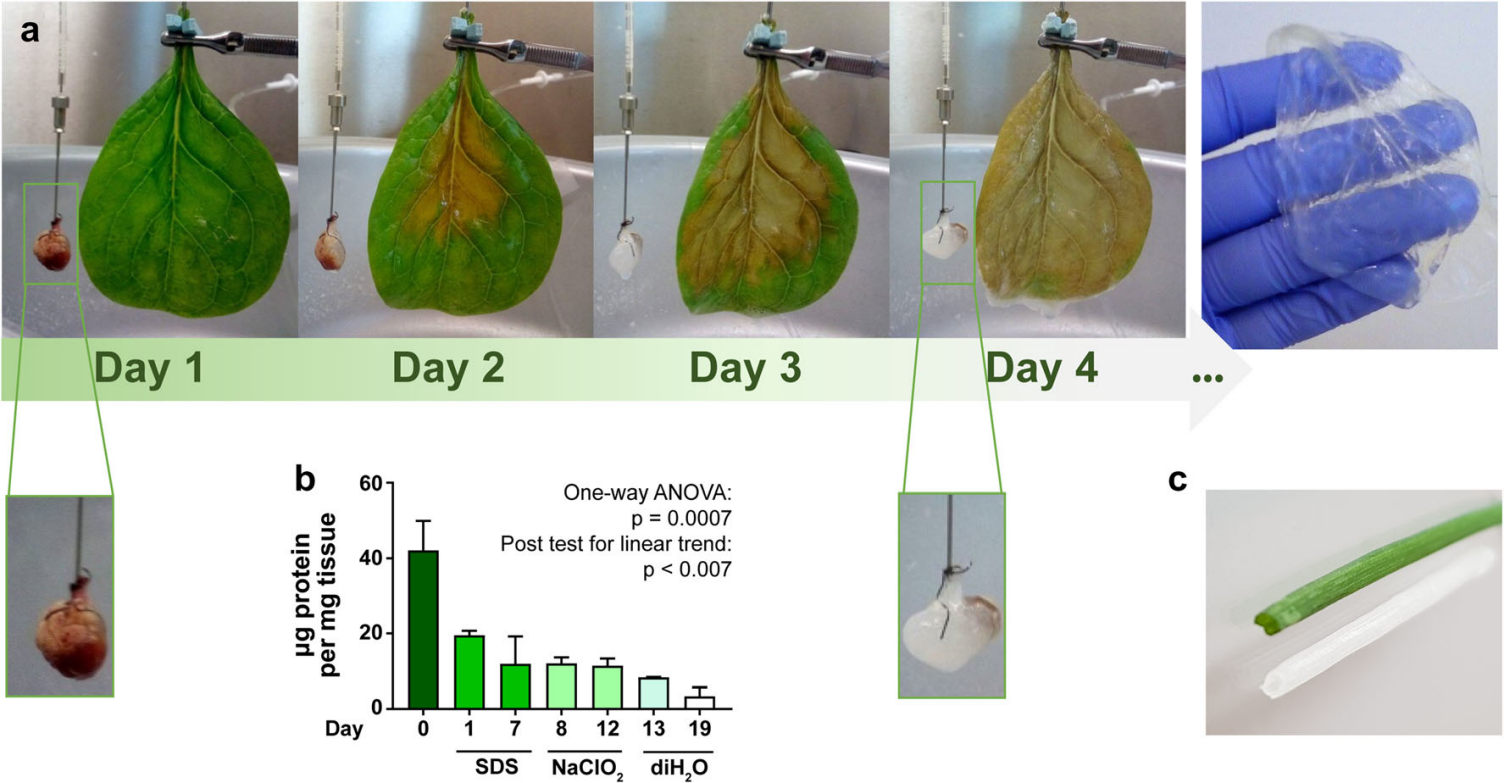
**Decellularization of a rat kidney, spinach leaf and chive.** **a** Decellularization process of a perfused spinach leaf next to a perfused rat kidney. **b** Protein quantification in chive samples at different steps of the decellularization process (*n* = 2). **c** Picture of native and decellularized chive

**Fig. 2:**
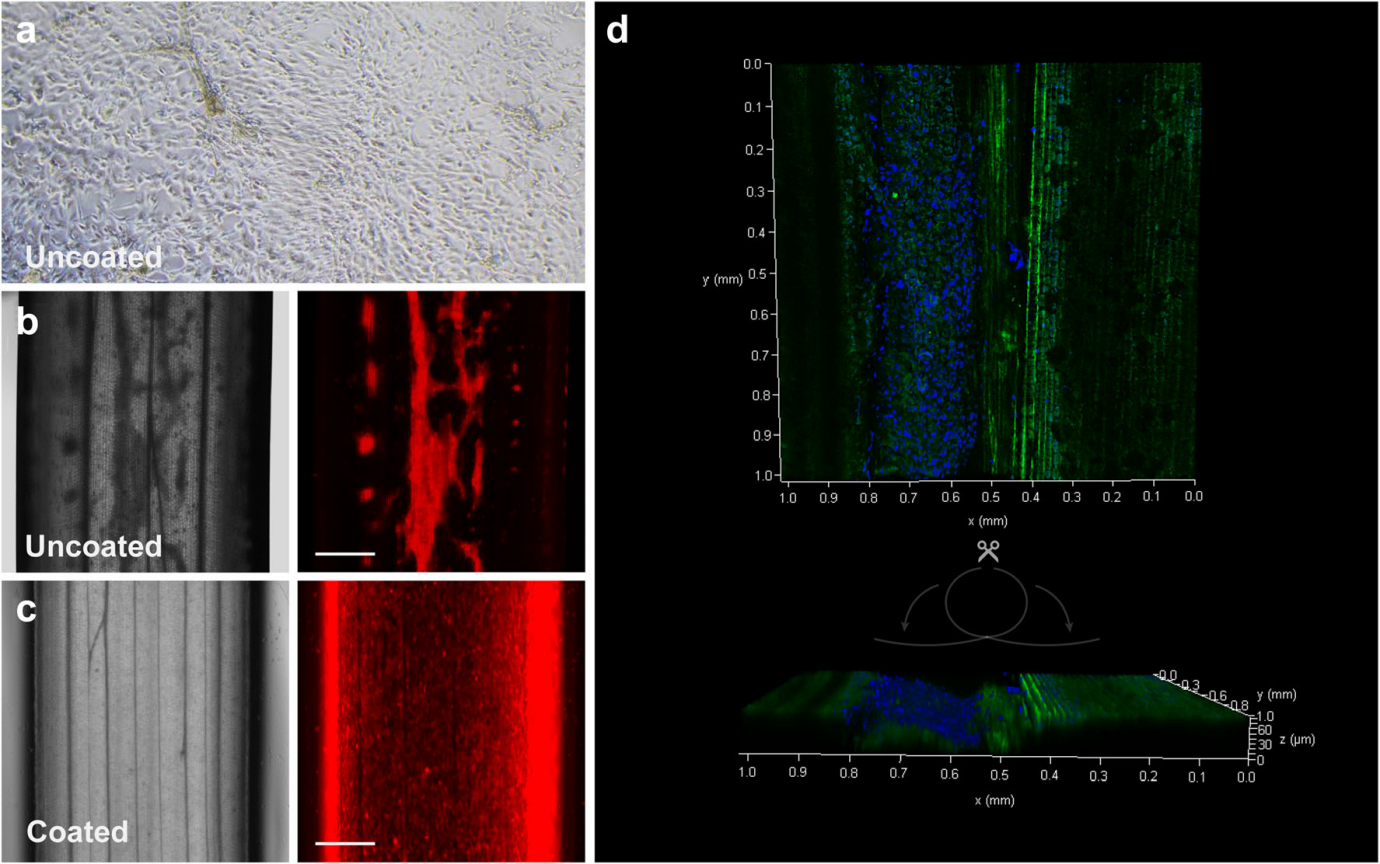
**Recellularization of chive-derived scaffolds with and without biofunctionalization.** **a** Representative brightfield picture of a 150 μm rat kidney slice 24 h culture after recellularization with ciPTEC. **b-c** Representative micrographs of decellularized uncoated and L-DOPA-coated chive with luminal seeding of TRITC-labeled ciPTEC (*n* = 3). **d** DAPI staining (blue) of ciPTEC 24 h after seeding in a cut and open-folded chive (autofluorescence green). Scale bars = 1 mm

We successfully applied the decellularization technique described by Gershlak *et al.* to produce plant-derived hollow tubular scaffolds from spinach leaves and chive stems (18). Decellularization by perfusion was much slower for spinach leaves than for the rat kidney, indicating less efficient penetration of the detergent (Fig. 1a). Moreover, decellularization through perfusion led to considerable SDS precipitation on the leaf, thus we proceeded with plate rocker incubation and minor adaptations in treatment lengths based on visual progress in decellularization (*i.e.*, 7 days 5% (v/v) SDS vs 5 days 10% (v/v) SDS, 5 days vs 2 days in sodium chlorite and Triton X-100 solution, and up to 7 days vs 2 days in deionized water). Similar to spinach leaves, we observed a gradual decrease in protein content in chive over the course of all washing steps (Fig. 1b). While cells attached to uncoated decellularized kidney slices (Fig. 2a), only few attached to the uncoated luminal chive scaffold wall. Instead, the cells grew in clumps that resided inside the lumen (Fig. 2b-c). Fontana *et al.* used biomineralization or coating with dopamine-conjugated tripeptide Arginine-Glycine-Aspartate (*RGD*) peptides for attachment of human mesenchymal stem cells and dermal fibroblasts (11, 18), but a simple coating with L-DOPA proved sufficient to create renal cell monolayers. Monolayers already formed 24 h after seeding but dominated one side of the lumen due to gravity (Fig. 2d). Extended culture time at 33 °C allowed cell proliferation and closure of the monolayer on the inner scaffold surface.

### Leaf Vasculature Appears to Have Anatomical Restrictions for Cell Infiltration

**Fig. 3:**
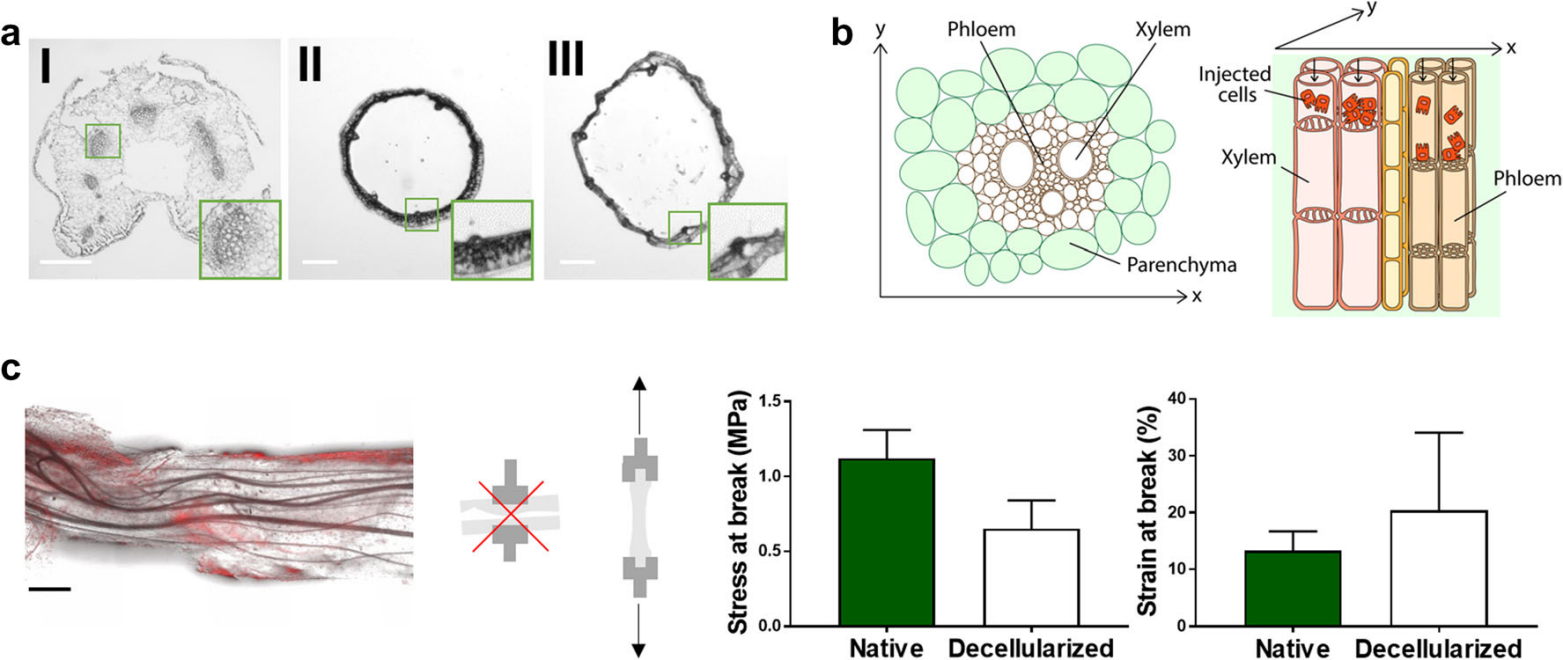
**Overview and anatomy of decellularized spinach and chive scaffolds for recellularization.** **a** Cross-sections of (I) decellularized spinach petiole, (II) native chive, and (III) decellularized chive, with zoom on vascular bundles. **b** Schematic illustration of plant vascular bundles and the assumed anatomical restriction of luminal recellularization. **c** After one week in culture, recellularized chive disintegrated along its fiber direction; stress and strain at break were only testable with tensile loading in longitudinal direction (*n* = 4). Scale bars = 1 mm

Renal cells could grow in the lumens of chive and spinach petioles, but they did not reach the spinach leaf vasculature through petiole injection. Despite the vascular recellularization claims by Gershlak *et al*., we believe that the plant vascular anatomy makes accessibility for cell seeding very unlikely. The petiole cavity is hollow and hence easily perfusable, but the vascular bundles, which form the conducting vessels for nutrients, water, and oxygen transport, are in fact located in the parenchyma next to the petiole cavity. Also in chive, the vascular bundles are located in the cortex, directly below the epidermis (Fig. 3a). The petiole cavities are air-filled, which fulfills three major functions: it saves growth energy, reduces tissue weight, and gives mechanical stability to enable growth in height and size. It is important to note that petioles are not connected to the leaf vasculature, which is separated from the parenchyma by impermeable structures to prevent water loss through diffusion. We suspect that, during decellularization, spinach leaves are not being perfused through the vasculature. More likely, the solution flows through the petiole cavity to enter the leaf parenchyma, either through diffusion or perfusion-induced breaks in the tissue. In accordance, the initial leaf discoloration pattern in Fig. 1a suggests general tissue penetration rather than selective vasculature perfusion. Apart from the lack of a direct connection between the petiole cavity and the leaf vasculature, a closer look at the anatomy of the vascular bundle itself, consisting of xylem and phloem, reveals another major hurdle for vascular recellularization (Fig. 3b). Xylem conducts water through tracheary elements, which are interconnected by areas that lack cell walls, known as perforations. Phloem transports soluble organic compounds through sieve tubes, which are also interconnected by perforations in their end walls to form transport channels (19, 20). These sieve-like areas likely trap injected cells. Even if our epithelial cells came through, they would grow over the perforations and hence occlude the vasculature (17). Gershlak *et al*. did not go into detail on the colonization of the inner surfaces with endothelial cells (10). From our results with epithelial cells, we conclude that recellularization of vasculature bundles in plant-derived scaffolds is unlikely obtainable and shifted focus to chive stems.

### Insufficient Scaffold Permeability for Transepithelial Transport Functionality

With chive as alternative tubular plant-derived tissue, we faced two other problems. First, although recellularization was performed successfully, both spinach and chive tissue quickly disintegrated in culture. Accordingly, it was not possible to apply traverse tensile loading on chive for mechanical testing; the tissue could only withstand tensile loading in longitudinal direction, along the cellulose fibers. Here, samples broke at quite deviating stress points, but stiffness and elasticity were in the same range as native tissue; stress at break was 0.7 ± 0.2 MPa compared with 1.1 ± 0.2 MPa for native tissue, and strain at break was 20 ± 13% compared with 13 ± 3% in native tissue (Fig. 3c). High variability between samples can be ascribed to the natural character of the scaffold source. The second problem was insufficient scaffold permeability, which is a requirement for rapid renal solute exchange between the inner and outer compartment through transepithelial processes (*i.e.,* passive diffusion and active transport). Earlier, we demonstrated the successful cellularization of biofunctionalized dialysis fibers, which allowed cellular transepithelial transport functionality (21, 22). Without cells, dialysis fibers leaked around 400 nmol FITC-inulin per min per cm^2^ when perfused with 0.1 mg/mL for 10 min (23). With decellularized chive, we did not measure any FITC-inulin leakage after 10 min.

## Discussion

**Fig. 4:**
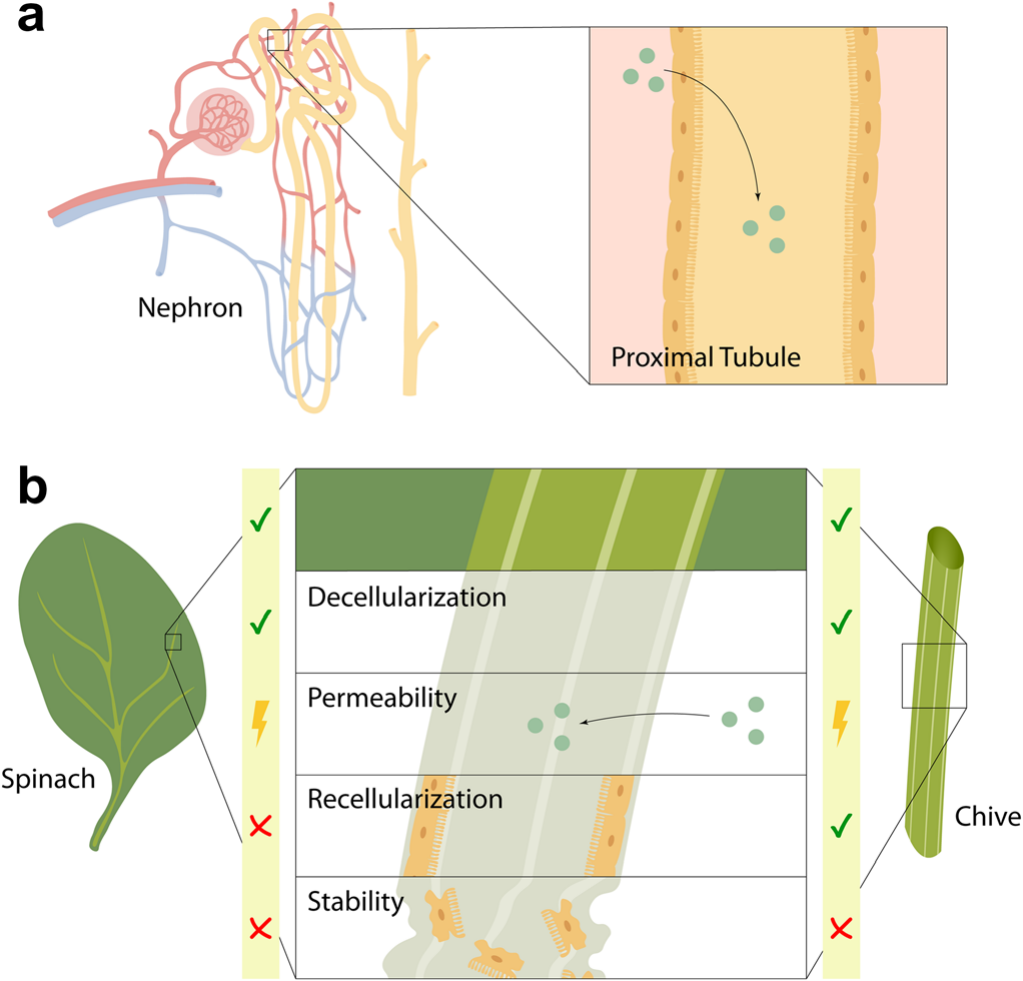
**Graphical overview of scaffold assessment for kidney tubule engineering.** **a** Nephron, the functional unit of a kidney with zoom into the proximal tubule. **b** Assessment of decellularized spinach and chive scaffolds for kidney tubule engineering

With their broad range of complex structures, decellularized plant scaffolds provide a natural and inexhaustible source for advanced *in vitro* modeling and regenerative organ replacement strategies. After biofunctionalization, all kinds of human cell types can be seeded onto the scaffolds as demonstrated by Modulevsky and colleagues in 2014, by Gershlak and colleagues in 2017, and by us in the work at hand (10, 12). However, tissue-specific scaffold requirements for functionality and implantability, if intended, need to be carefully considered. In case of kidney tubule engineering, a thin scaffold sheet with complex tubular vasculature and a joint drainage, as provided by spinach leaves, would have met important anatomical requirements. Unfortunately, injected cells did not reach and distribute within the leaf vasculature, probably due to anatomical restrictions. Moreover, while we confirmed the general compatibility of decellularized plant scaffolds and kidney cells, the tissue-specific requirement of permeability for rapid solute diffusion was not met. Therefore, we consider neither spinach leaves nor chive as suitable for functional kidney tubule engineering. In addition, the observed tissue disintegration in culture suggests inappropriateness for implantation. An overview of our assessment is given in Fig. 4. Modulevsky* et al*. provided evidence for biocompatibility and implantability for apple-derived cellulose scaffolds, which are mainly composed of cell walls that contain pores and air pockets that facilitate cell invasion, the transport of nutrients and water, and vascularization *in vivo* (13). Next steps in this research group included scaffold examination for bone tissue-specific applicability (not yet peer-reviewed (24)). Moreover, very recently the US Food and Drug Administration recognized CelluBridge™, a plant-based spinal cord scaffold implant, as a Breakthrough Medical Device. While sponge-like plant-derived scaffolds might provide a suitable architecture for bone and spinal cord tissue, it is not suitable for kidney tubules that require scaffold compartmentalization and drainage capacity. With this paper, we want to highlight that unusual solutions often generically sound attractive but nevertheless require careful examination for purpose-specific evidence.

Despite limitations of plant leave structures for regenerative medicine due to likely tissue disintegration, drug development might still be able to benefit from engineered test models. With the provision of relatively low material stiffness, high water content, 3D structure and topographic guidance, cell lines can be cultured under more physiologically relevant conditions, which could translate into more predictive results. Insufficient scaffold permeability for renal transepithelial transport functionality does not exclude suitability for other cell models, such as endothelial cells and human pluripotent stem cell derived cardiomyocytes, which have shown normal functioning on decellularized spinach leaves (10). With regard to kidney tubule engineering, perhaps the combined use of plant-derived scaffold materials and other techniques could open doors for advanced *in vitro* modeling. Current technologies such as 3D printing, molding, and weaving could be used to integrate plant-derived structures and biomaterials for enhanced tissue engineering capabilities (25). For example, He *et al*. developed a microreplication method to transfer the microvascular network of leaf venation into hydrogels (26). This could be one way to take advantage of the complex plant structure while circumventing the limiting properties that we revealed with the work at hand. The various possibilities for the use of plant-derived and other unconventional biomaterials have been reviewed elsewhere (27).

## Conclusion

Although plant-derived cellulose scaffolds can be recellularized with mammalian cell types, tissue-specific requirements must be considered for proper tissue engineering. Here, we showed that decellularized spinach leaves and chive are unsuitable for kidney tubule engineering due to micro-anatomical limitations in the leaf venation for recellularization and limited scaffold permeability as premise for transepithelial solute exchange. Plant-derived biomaterials have still potential for advanced cell culture but need to be carefully selected and tested for tissue-specific functionality.
